# Automating mosquito taxonomy by compressing and enhancing a feature fused EfficientNet with knowledge distillation and a novel residual skip block

**DOI:** 10.1016/j.mex.2023.102072

**Published:** 2023-02-10

**Authors:** Francis Jesmar P. Montalbo

**Affiliations:** College of Informatics and Computing Sciences, Batangas State University, Batangas City, Batangas, Philippines

**Keywords:** Deep learning, Convolutional Neural Networks, Transfer Learning, Fine-Tuning, Feature Fusion, Residual Learning, Efficientnet, Entomology, Compression and Enhancement of an Automated Deep Learning model for Mosquito Taxonomy

## Abstract

Identifying lethal vector and non-vector mosquitoes can become difficult for a layperson and sometimes even for experts, considering their visual similarities. Recently, deep learning (DL) became a solution to assist in differentiating the two mosquito types to reduce infections and enhance actions against them. However, the existing methods employed to develop a DL model for such a task tend to require massive amounts of computing resources and steps, making them impractical. Based on existing methods, most researchers rely on training pre-trained state-of-the-art (SOTA) deep convolutional neural networks (DCNN), which usually require about a million parameters to train. Hence, this method proposes an approach to craft a model with a far lower computing cost while attaining similar or even significantly better performance than pre-existing models in automating the taxonomy of several mosquitoes. This method combines the approach of layer-wise compression and feature fusion with enhanced residual learning that consists of a self-normalizing activation and depthwise convolutions.

• The proposed method yielded a model that outperformed the most recent and classic state-of-the-art deep convolutional neural network models.
• With the help of the modified residual block and knowledge distillation, the proposed method significantly reduced a fused model's cost while maintaining competitive performance.
• Unlike other methods, the proposed method had the best performance-to-cost ratio.

## Graphical abstract

**Figure:**
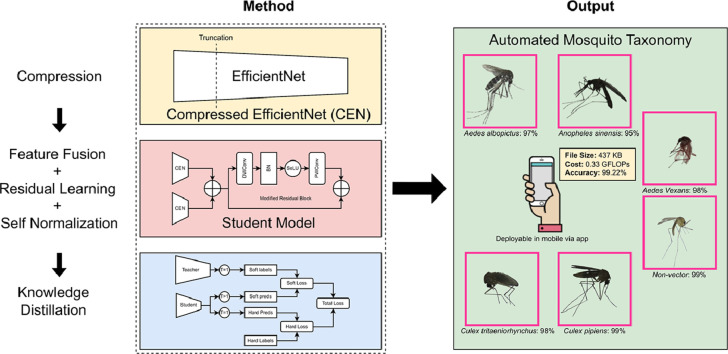

Specifications table

**Table:** 

Subject Area	Computer Science

More specific subject area:	*Deep Convolutional Neural Networks and Entomology*
Method name:	Compression and Enhancement of an Automated Deep Learning model for Mosquito Taxonomy
Name and reference of original method:	This proposed method has several techniques combined, cited accordingly within the method article.
Resource availability:	The resources needed to reproduce this article can be found on the link below. The link provides all source codes and the dataset. https://github.com/francismontalbo/mosquito_kd_2021

## Method details

In our advancing world, various disciplines deem Deep Convolutional Neural Network (DCNN) models as one of the leading solutions to solve problems automatically. Based on most research, DCNN models have shown tremendous performance in doing classifications across various image data [1]. However, such benefits come with a price, as most DCNN models performing non-trivial tasks with limited data tend to rely on large and complex architectures. These traits made DCNNs challenging to produce and deploy in some areas with inadequate computing power [2]. The reason for DCNNs’ lengthy and broad network architecture lies in their goal of classifying thousands of classes with millions of images [3]. However, based on recent research papers, most researchers only use DCNN models to classify samples below the said numbers, with only a few classes of <100. In some cases, they even use them for binary classifications with only a few samples of <10 K per class. Hence, making them considerably costly for such tasks [4], [5], [6], [7], [8].

Currently, one of the most used methods to reduce the cost of DCNNs and make them operate with custom datasets is via transfer learning (TF) and fine-tuning (FT) [9]. TL transfers specific pre-trained weights from the ImageNet dataset to a specific DCNN. In common practice, DCNNs that receive pre-trained weights require FT to make them accustomed to the dataset of choice. As observed in most recent studies, DCNN models that acquired TL and FT specifically for the identified task usually show better performance even with fewer parameters. The reduction occurs due to the irrelevant upper layers or head and neural network layers extracted that contain the previously labeled weights from ImageNet [10]. Though TL and FT solved the problem of training DCNN models for a specific task with less cost, most still tend to consist of millions of parameters [11]. Due to DCNNs’ recent popularity and ability to perform automated classifications, research studies began to utilize them to decipher challenging tasks correlated to mosquito taxonomy. In one study, Park et al. used a DL model to mechanize the taxonomy of six classes of mosquito species [12]. Their study trained a state-of-the-art (SOTA) DCNN model called VGG16 using a portion of their mosquito dataset of ≈3600 images. According to their results, their VGG16 model attained an accuracy of 97.74%. Though they achieved such a feat, their model required about 138 M parameters to train, making it relatively inefficient for low-end devices. On the other hand, though they considered cheaper models like ResNet50 with 25 M and SqueezeNet with 1.23 M parameters, they had lower accuracies, as they only attained 96.86% and 90.71%, respectively. Fortunately, other researchers set out to study how they can further decrease the cost of DCNN models without sacrificing a substantial fraction of their performance toward a particular task.

In a distinguished study by Das et al., aside from TL and FT, they further reduced the cost of their DCNN model by trimming some of its layers. Upon evaluation, they observed that their selected DCNN model, InceptionV3, which had lesser parameters and layers after truncation, still performed satisfactorily side-by-side with a typical FT InceptionV3. In conclusion, they discovered that vast and complex DCNN models do not wholly need all their layers when training with smaller datasets than ImageNet [13]. In the following study, Montalbo, F. J. P., also had a model condensed but at the same time fused to supply additional features that can boost performance despite the reduced feature-generating layers. The study revealed that a layer-wise fusion of features effectively increases the number of features without extending the number of parameters in the DCNN model [14]. Though the mentioned studies shrank the length and expense of DCNNs, they did not employ other possible compound advances to expand a DCNN's performance further.

With the identified problems and existing methods mentioned regarding cost reduction, this method proposes to craft a less costly DCNN model that can run radically better than most existing solutions. Like Park et al.’s study, this method aims to automate the taxonomy of mosquitoes, including lethal vectors and non-vector. This method can assist laypersons and even experts in identifying mosquitoes correctly without needing a cumbersome model. Offering such can flourish awareness, avert unwanted infections, and better actions toward protection and extermination. Aside from usual TL, FT, feature fusion, and model compression, it is worth mentioning that this method invokes Knowledge Distillation (KD), self-normalization, and Depthwise Convolutions (DWConv), expounded in subsequent sections of this article.

## Improving cost-efficiency

The first step of the proposed method focuses on building a compact model that will receive the distilled knowledge from a more cumbersome teacher model about various mosquito classes. However, due to the vast possibilities and undefined approach to finding the best teacher model, this method reviewed well-known state-of-the-art (SOTA) DCNN models that suit this method's needs. Upon review, it shows that EfficienNet embodies the most relevant characteristics of the proposed method.

**Fig. 1:**
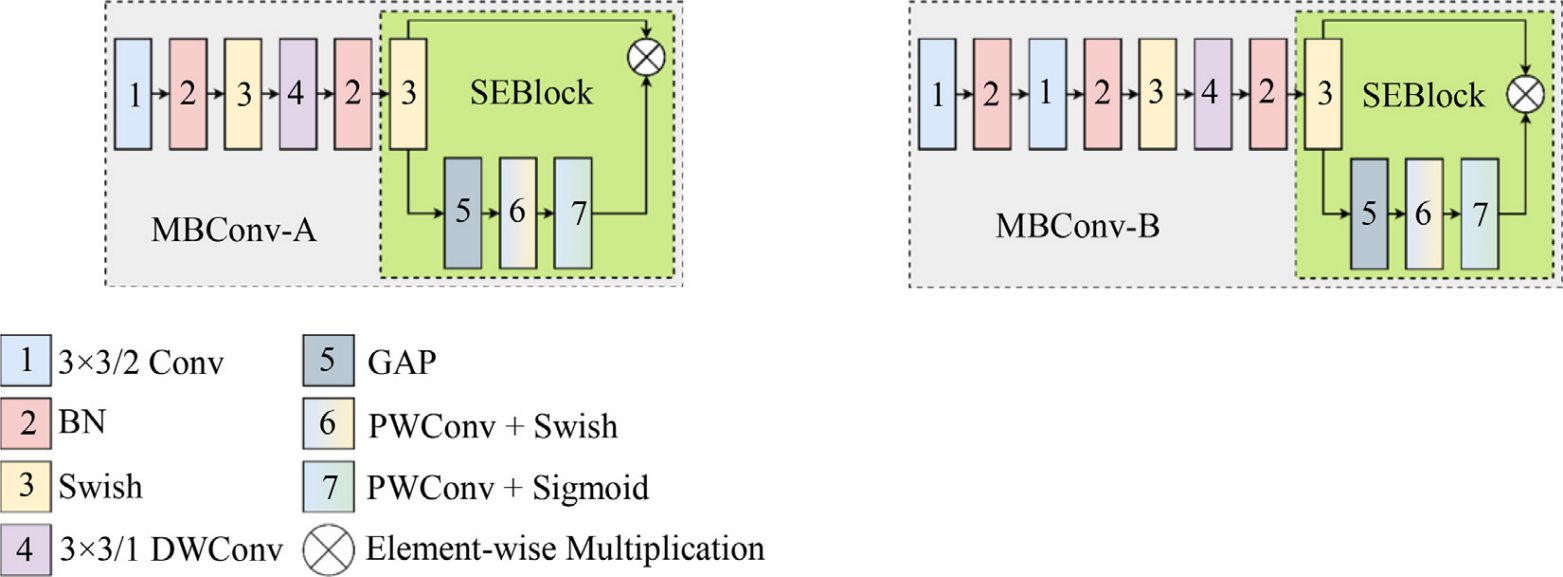
Inverted bottleneck residual block or MBConv of EfficientNet.

According to the specification of EfficientNet, its structure focuses on employing lighter convolutions (Conv) in the form of an inverted bottleneck residual block or MBConv, equipped with a squeeze-and-excitation block (SEBlock) [15], [16]. Fig. 1 illustrates the said MBConv with two versions used by the EfficientNetB0. Based on the figure, it contains a series of layers that produces the *x* features using a specific *k* × *k* kernel that convolves over a *H* × *H* sized image, divided by a specific stride value of /2. The following includes a 3 × 3/2 Conv, Batch Normalization (BN), Swish activation function, and 3 × 3/1 DWConv, arranged in the given order. The upper layers of an MBConv-A have a connection pattern of a 3 × 3/2 Conv → BN → Swish → 3 × 3/1 DWConv → BN → Swish that connects to the SEBlock. The SEBlock uses a skip connection composed of Global Average Pooling (GAP) [17], a 1 × 1/1 Conv, or a Pointwise Conv (PWConv) activated by Swish [18] and another PWConv with sigmoid [19]. These last layers then enter an element-wise multiplication ⊗ together with a Swish activation function.

The base EfficientNetB0 model, or the lightest in the family of EfficientNets, roughly consists of 5 M parameters. As stated, it can effortlessly scale based on its feature depth and spatial dimensions, shifted by incrementing its composite coefficient [20]. However, considering its cost-efficiency, the given parameters can still become costly at certain times. Therefore, this method proposes employing a layer reconstruction method to remove most of its layers and produce a compressed version. It is worth mentioning that other sections of this proposed method will provide the handling of certain drawbacks caused by this truncation method.

**Fig. 2:**
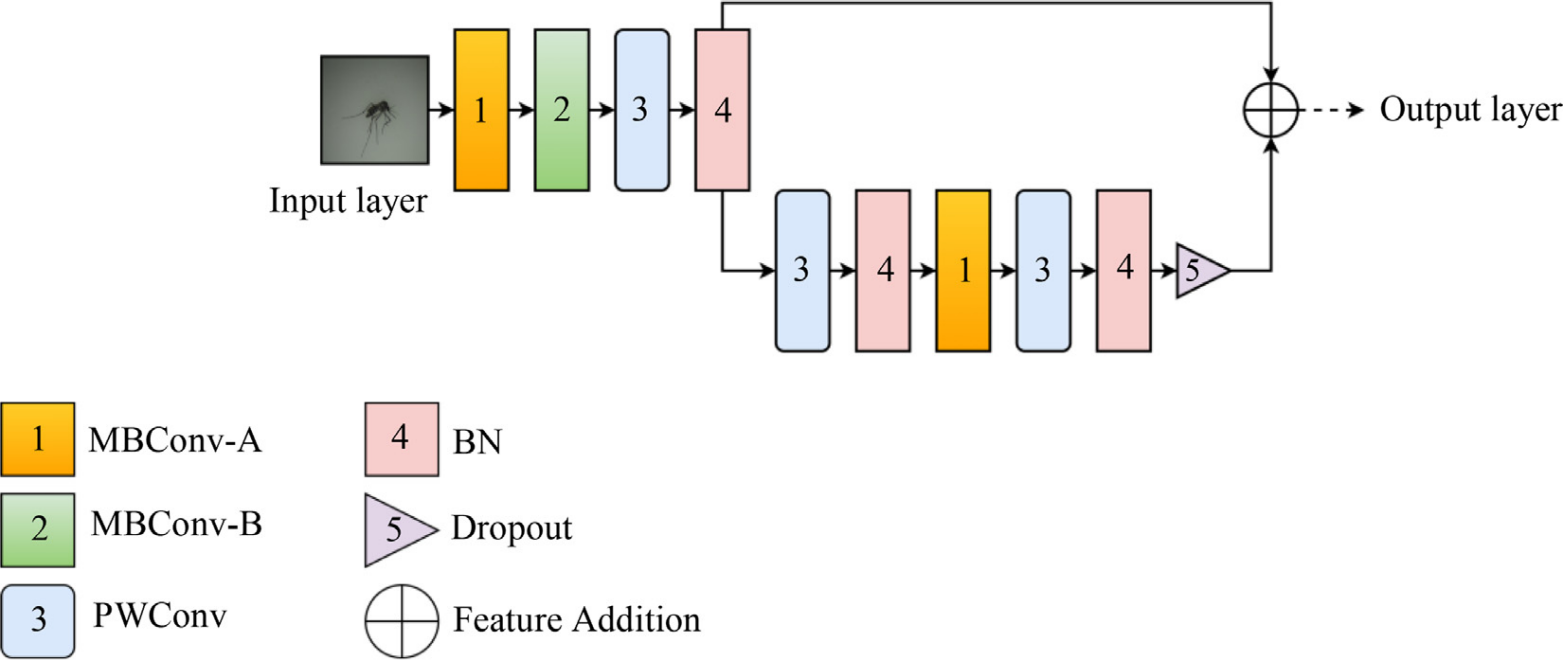
The Compressed EfficientNet (CEN) architecture.

As illustrated in Fig. 2, the compressed EfficientNet (CEN) architecture takes only the core entry block of the original EfficientNetB0, making it far lighter and shorter than its original form but still retains its initial core feature generating layers, the MBConvs. After the layer compression, the initial parameters of the EfficientNetB0 went down from 5 M to only 20 K, showing a drastic change in complexity and cost.

## Expanding features

**Fig. 3:**
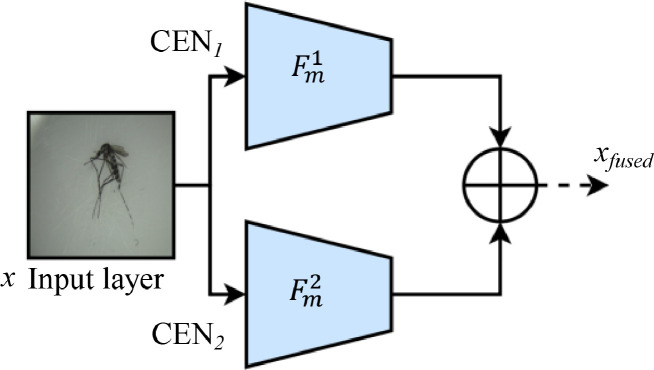
The fused Compressed EfficientNet (FCEN) architecture.

Considering the reduced number of layers, CEN can experience an adverse effect of reduced performance. Hence, the second step of this method follows with a feature fusion to re-increase the depth of features without re-elongating the end-to-end network architecture, as illustrated in Fig. 3.

Based on the illustration, a CEN*_m_* model takes an input *x* from an image with a *H × H* spatial dimension to produce its *F_m_^u^* features. As mentioned, these features can become insufficient after compression due to the fewer layers that handle them. In this method, feature fusion became a way to alleviate this problem. The proposed method had a mirror CEN*_m_* model that stochastically generates another set of *F_m_^u^* from the same *x* input, yielding *F^1^_model_* and *F^2^_model_*. In Eq. (1), feature fusion occurs by having an element-wise addition function ⨁, which adds both feature sets to produce a new set of *x_fused_* inputs from the fused CEN (FCEN) for the next layer [21].

(1) $$x_{fused}=F_{model}^{1}\left(H\times H\right)⊕F_{model}^{2}\left(H\times H\right)$$

## Implementing skip connections

**Fig. 4:**
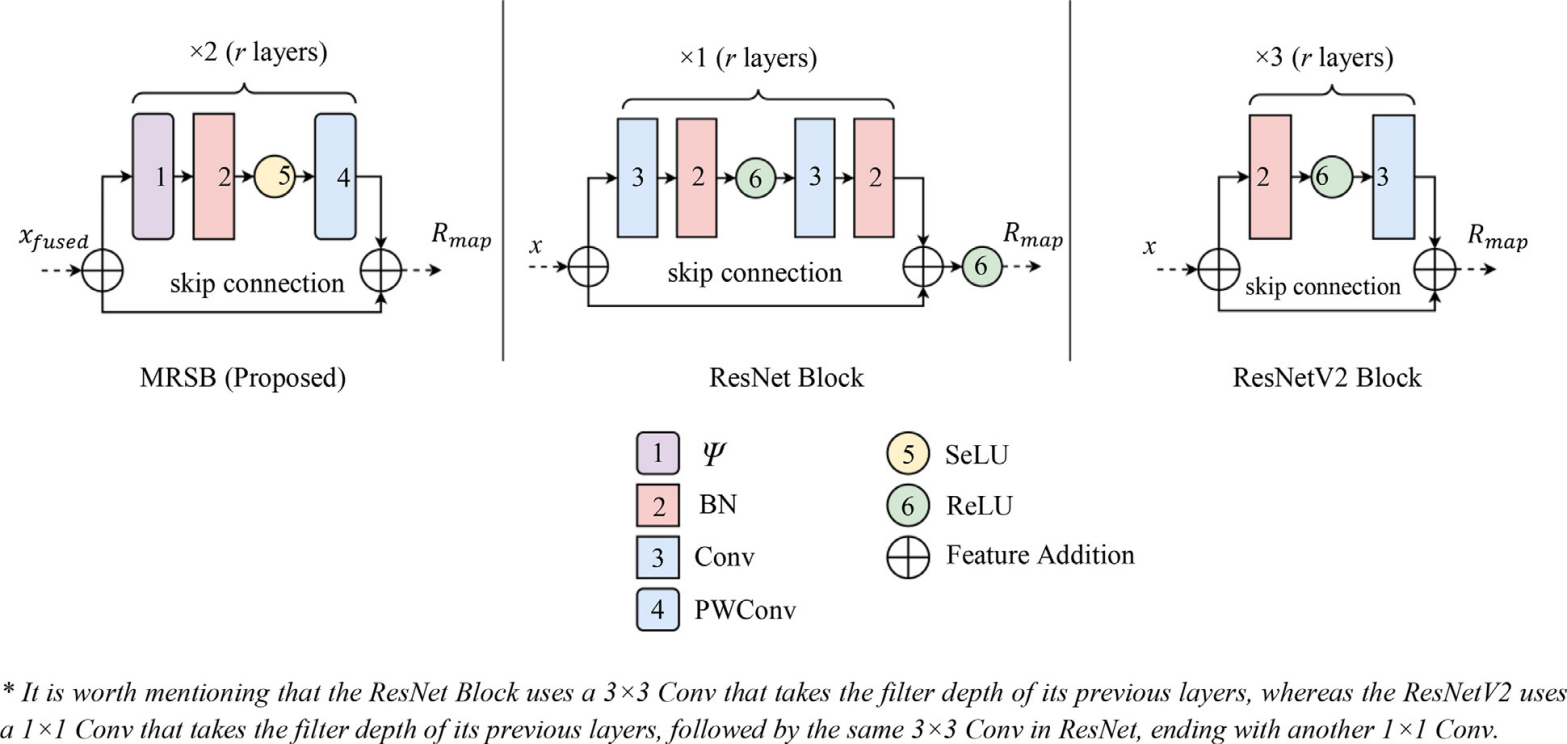
The modified residual skip block (MRSB) compared with the ResNet blocks.

The inadequacy of data and the robustness of fused features can potentially lead to overfitting [22]. Therefore, the proposed method also considered residual learning to handle the fused features *x_fused_* from the FCEN model to alleviate the problem and simultaneously produce better performance [23]. Fig. 4 illustrates the modified residual skip block (MRSB) of the proposed method side-by-side with the original ResNet and ResNetV2 blocks for differentiation. Unlike ResNets, which uses the standard rectified linear units (ReLU) as activations within its residual mechanisms, this proposed method utilizes SeLU to integrate self-normalizing properties into the fused network [24]. In addition, due to the expense of multi-stacked Conv layers. This method relied on a lighter 1 × 1 DWConv layer with a depth of 1 or Ψ, defined in the following equations. The altered arrangement also aims to provide better gradient flow while being more cost-efficient.

Based on the presented adaptation of an MRSB, the following explains additional details about its purpose and how it can deliver better performance with less costly production. Eq. (2) indicates how the residual map *R_map_* gets produced. As denoted, the {*ω_j_*} weighted *r* layers within the MRSB produce the *R_map_* using a residual function ℱ [25].

(2) $$R_{map}=F\left(r,\left\{\omega_{j}\right\}\right)+r$$

## Adding self-activating layers

**Fig. 5:**
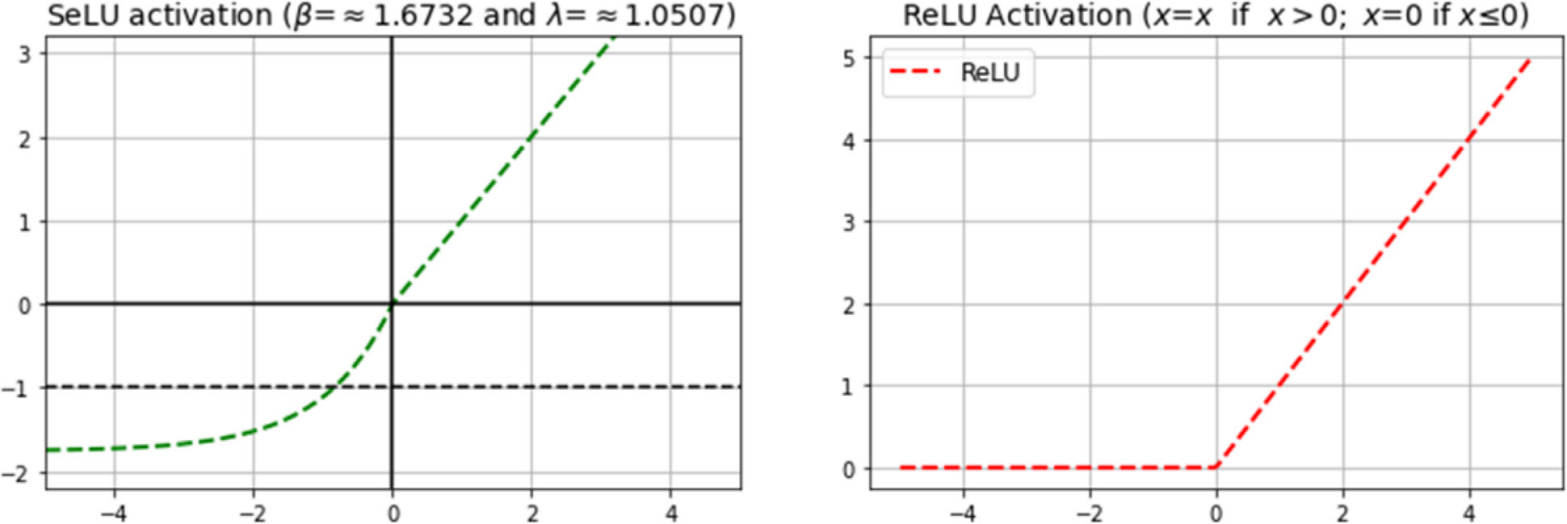
Difference between SeLU (a) and ReLU (b) activation plots.

In (3), the ℱ function activates the weighted *ω_j_r* layers with SeLU, visualized in Fig. 5(a), to produce the desired *R_map_*, where the proposed method used the SeLU activation as its core activation function.

(3) $$F=\omega_{j}SeLU\left(\omega_{1}r\right)$$

For better reasoning as to why this method used SeLU, (4) defines the ReLU activation function based on a simple function ReLU=max(0, *x*). According to the piecewise function, ReLU bases its actions on a feature's value, whether it gets maxed out to a non-zero value or reduced to a zero value, which can sometimes lead to a loss of information [26].

(4) $$ReLU\left(x\right)=\left\{ \begin{array}{c} x=x,\;if\;x>0\\ x=0,if\;x\leq 0 \end{array}\right.$$

ReLU, as shown in Fig. 5(b), recently built its credibility in DL due to its performance against the “*vanishing and exploding”* gradient problem. However, ReLU does not include self-normalization properties and regularization, making it prone to the mentioned problem if the model does not receive enough feature values due to the lack of data. Therefore, SeLU became the choice for this method, as it tends to accommodate a small dataset with < 10 K samples and has a shorter set of processes to produce more features. In (5), SeLU uses constant parameters β=≈1.6732 and λ=≈1.0507 that handle the self-normalization of features and preserve their variance to a [0, 1] range [27]. Such an approach strongly regularizes the flowing gradients compared to a ReLU function while preventing information loss.

(5) $$SeLU\left(x\right)=\lambda \left\{ \begin{array}{c} x=x,\;if\;x>0\\ x=\beta exp^{x}-\beta ,if\;x\leq 0 \end{array}\right.$$

## Reducing parameters

This section exemplifies the difference between conventional Conv layers and DWConv layers. Considering that the proposed method incorporated an MRSB, its composition is one of its cost-reducing factors. As previously shown, the MRSB does not rely on a typical Conv layer. Instead, it generates the *R_map_* with DWConv and a PWConv in its *r* layers.

Eq. (6) presents how a Conv layer produces feature maps within a model *F^u^_model_*. Having an *x* input with an equal spatial dimension of *H_F_^2^* and *C* takes in a *k × k* kernel *K* that convolves with a specific stride value of ≥1. After completing the Conv process over the entire network, a Conv feature *H_K_ × H_K_ × C × C^’^* gets produced as *Conv_out_,* where *C’* represents the output channel [28].

(6) $$Conv_{out}=\sum K\left(C,C^{\prime }\right)\times F_{model}^{u}$$

Considering the previous equation, (7) presents how complex and costly Conv layers could become [28].

(7) $$H_{K}^{2}\times C\times C^{\prime }\times H_{F}^{2}$$

Based on how the Conv operation produces a feature map, the DWConv splits the operation into two sections. First, the DWConv performs its channel-wise extraction with a PWConv that uses *Pw*, which serves as its *K* with an equal spatial dimension of 1. The channel-wise features then get stacked in as a 3D tensor. Secondly, the DWConv summates all the extracted channel-wise features (8). This approach permits the DWConv to capture pointwise features with fewer calculations when producing feature inputs but requires additional processing time.

(8) $$DWConv_{C=}\sum K\left(Pw,\;C\right)\times F_{model}^{u}$$

Due to the reduced calculations from the DWConv, (9) shows how it simplified and lessened the cost of producing feature maps. Based on a study, the DWConv can reduce a Conv layer's cost by ≈ × 9 [28], [29].

(9) $$\frac{\left(H_{K}^{2}\times C\times H_{F}^{2}\right)+\left(C\times C^{\prime }\times H_{F}^{2}\right)}{H_{K}^{2}\times C\times C^{\prime }\times H_{F}^{2}}=\frac{1}{C^{\prime }}+\frac{1}{H_{F}^{2}}$$

**Fig. 6:**
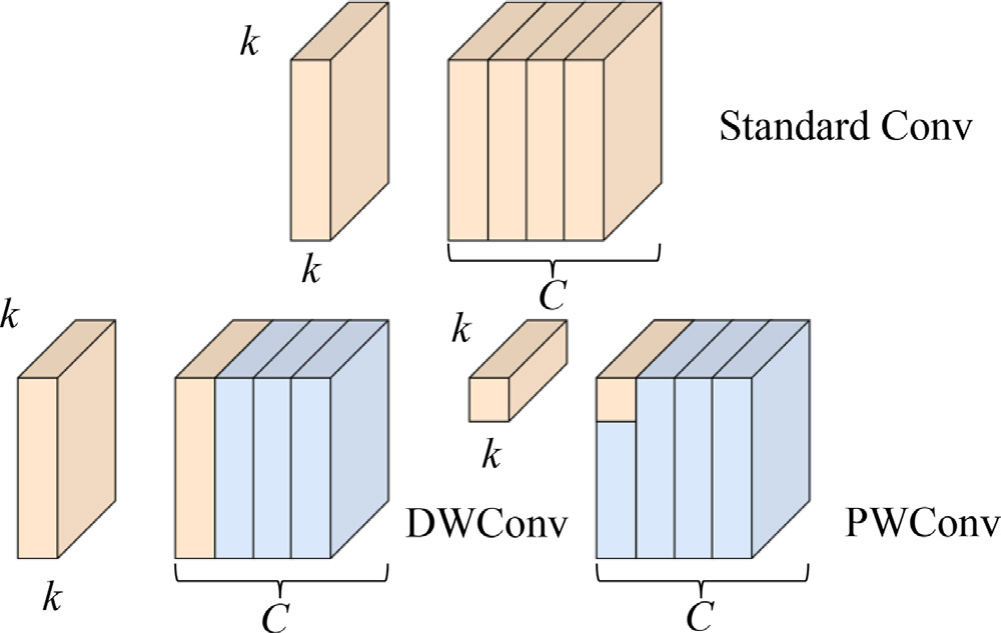
Differences between standard, depthwise, and pointwise convolutions.

From a more visual standpoint, Fig. 6 presents the differences between a Conv, DWConv, and PWConv layer. Observably, the Conv layer focuses on extracting features from the entire image dimension and its depth, while DWConv only focuses on depth or filter. On the other hand, the PWConv only uses a 1 × 1 *K* to go over the entire spatial dimension of the image, conserving parameters but may take more extended periods to finish.

## Transfer learning and fine-tuning

Due to the core architecture of this method being EfficientNet, TL became possible. As mentioned, TL provides the model an added leverage to learn pre-trained features from the ImageNet database. However, learning those features can delineate the model away from the target mosquito classes. Therefore, FT became a vital factor in taking advantage of the pre-trained weights to become of use.

**Fig. 7:**
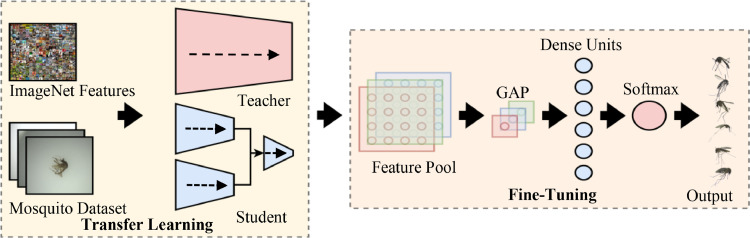
Transferring ImageNet weights and fine-tuning the network to learn the mosquito classes.

As illustrated in Fig. 7, this proposed method had both CEN*_1_* and CEN*_2_* receive the pre-trained weights separately from ImageNet via TF. Together with FT, both models had additional layers, including a GAP, dense neurons of 6 representing the classes of interest, and a softmax activation to extract the initial logits needed for KD. On the other hand, the teacher model also received the same treatment when it had its logits extracted for KD.

## Distilling knowledge

In this proposed method, the FCEN with an MRSB did not solely undergo the conventional training approach. Instead, it received distilled knowledge via KD from a SOTA teacher model with the highest overall accuracy in identifying the mosquito species from the source dataset.

During KD, the process utilizes a modified softmax *Q* shown in (10). As denoted, the function incorporates a temperature parameter *τ,* where if a student uses the modified softmax with a value >1, it generates the student's logits *L_s_*. On the other hand, setting the τ value to 1 returns the softmax function to its original state that generates the teacher's logits *L_t_* [30], [31]. This proposed method used a value of τ >1 when conducting KD and a value of 1 during FT.

(10) $$Q\left(\tau \right)=\frac{exp\left(L_{t}/\tau \right)}{\sum_{s}exp\left(L_{s}/\tau \right)}$$

After both models had produced their logits, they also generated their respective predictions. The teacher model used *L_t_* to produce its predictions, referred to as soft labels *θ_i_*. At the same time, the student used *L_s_* to generate its soft predictions *δ_i_*. Due to the student training with a standard softmax τ=1, using *G* samples and labels from the prepared mosquito dataset, the proposed method produces the hard predictions *P*.

However, for the KD model to produce its final predictions, it requires the total loss *Total_loss_* from both the teacher and student. Therefore, the teacher and student must first produce their respective loss scores using specific loss functions to produce the final predictions. KD refers to these losses as soft loss *Soft_Loss_* and hard loss *Hard_Loss_* [32].

In (11), the teacher model uses the Kullback-Leibler loss function (*KL_Loss_*) [33] to define the difference between the predictions from the teacher's *θ_i_* and the student's *δ_i_* to produce *Soft_Loss_*. The *N* denotes the number of classes, and *i* denotes the first label instance of the mosquito dataset.

(11) $$Soft_{Loss}=\sum_{i=1}^{N}KL_{Loss}\left(\theta_{i},\delta_{i}\right)$$

To produce the *Hard_Loss_* in (12), the student model takes the predictions *P* and maps it with ground truth labels *G* produced from the modified softmax τ>1. The *Hard_Loss_* uses a standard categorical-cross entropy loss *CCE_Loss_* [34] with labels set to >2, as the proposed method has six classes.

(12) $$Hard_{Loss}=\sum_{i=1}^{N}CCE_{Loss}\left(P_{i},G_{i}\right)$$

With both losses produced, the KD model computes the *Total_loss,_* generating the final predictions. The *Total_loss_* utilizes a specific balancing parameter *α* to adjust the weights between the teacher and student, reducing the superiority of one model over the other. In (13), it shows that the *Total_Loss_* is the sum of the weighted *Soft_Loss_* × (1-*α*) and the *Hard_Loss_ × α.*

(13) $$Total_{Loss}=Soft_{Loss}\times \left(1-\alpha \right)+Hard_{Loss}\times \alpha$$

**Fig. 8:**
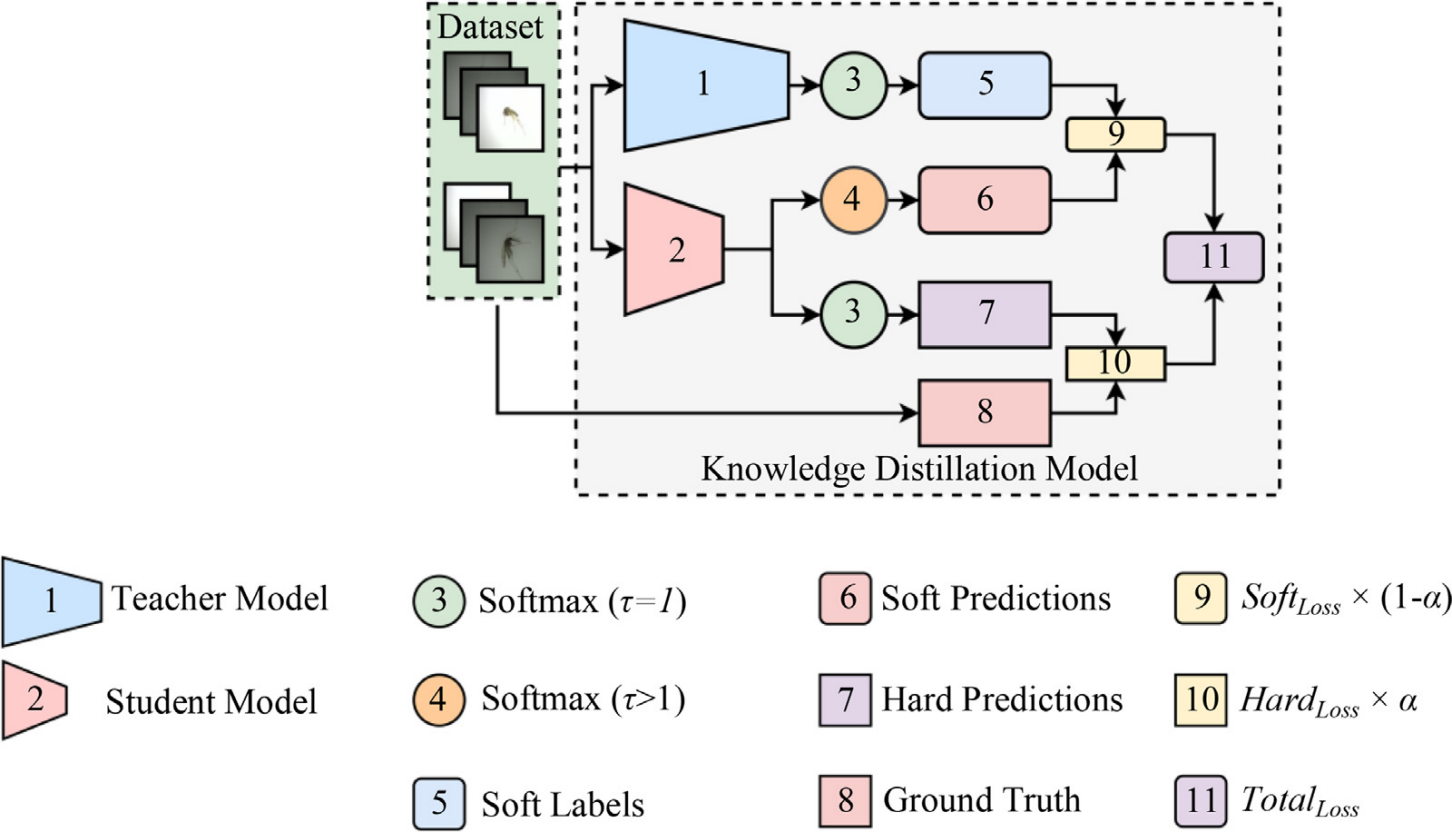
The knowledge distillation approach.

For a better overview, this method illustrates the KD process in Fig. 8. The process begins by training the teacher model with the prepared mosquito dataset using a standard softmax function to produce the soft labels. On the other hand, the student trains with the modified softmax function with a *τ*>1 and a standard softmax with *τ*=1, producing soft and hard predictions, respectively*.* Both models then calculated the losses between their ground truth labels and predictions using the defined loss functions, producing the *Total_Loss_* or the final predictions of the KD model.

## Method validation

As mentioned, the proposed method aims to deliver a better solution by having a lightweight model that can accurately classify specific mosquito species. However, the method must present validatable results to justify whether it achieved such a feat. Therefore, in Table 1, using commonly used metrics like accuracy, precision, recall, and f1-score, this method calculates and compares the performance of the produced model against well-known SOTA models [35].

**Table 1:** Evaluation metrics.

Metric	Equation	Description
Accuracy (*Acc*)	(TP+TN)/(Allsamples)	Calculates the ratio of all positive and negative predictions over all samples to identify a model's overall performance.
Precision (*Pr*)	TP/(FP+TP)	Measures a model's ability to count TP genuinely
Recall (*Rc*)	TP/(FN+TP)	Identifies a model's capacity to justify between positives from actual positives.
F1-Score (*F1*)	2×(Pr×Rc)/(Pr+Rc)	Combines the scores of *Pr and Rc* to calculate the harmonized mean.

In addition, this article also presents the calculated Floating-Point Operations per second (FLOPs) to highlight the cost-efficiency of the model produced by this method. Eq. (14) presents the FLOPs calculation for the upper feature extraction layers of the model [36].

(14) $$FLOPs=H^{2}\left(C\times K+1\right)C^{\prime }$$

On the other hand, (15) presents the remaining dense connections and classifier, where *I* denote the calculated upper input layers up to the output layers *O* [36].

(15) FLOPs=(2I−1)O

For measuring the model's performance validity, this method used an open-sourced dataset by Park et al. that contains about 3600 images of mosquitoes classified into five classes [12]. As specified in Table 2, this method followed the Pareto principle of having 80% of the entire dataset for training, whereas 20% for validating its performance.

**Table 2:** Specification of the mosquito dataset used by the proposed method.

Class	Train Qty. (80%)	Validation Qty. (20%)	Total Qty (100%)
Non-Vector	480	120	600
*Aedes albopictus*	480	120	600
Aedes vexans	473	118	591
Anopheles sinensis	485	108	593
Culex pipiens	420	180	600
*Culex tritaeniorhynchus*	475	119	594
**Total**	2813	765	3578

For ease of reproduction, Table 3 presents the following hyper-parameters used in this method. Since KD requires a teacher model, this method trained a list of teacher candidates beforehand. In addition, the student model trained with a standard softmax also used the presented hyper-parameters. It is worth mentioning that the values can differ depending on the machine. Though arbitrary, the main factors considered for the selected hyper-parameters lie in the current machine specification of this method that had an RTX 3060 12GB and their commonality with most studies [37].

**Table 3:** Hyper-parameters settings for training the teacher candidates and a non-KD student.

Hyper-parameter	Value
Batch Size	16
Optimizer	Adam
Epochs	30
Learning Rate	0.0001

Apart from the given hyper-parameters, this method also had additional hyper-parameters specifically for KD, as shown in Table 4. During KD, the model performs another training or a distillation stage using the previously presented hyper-parameters but with a different learning rate. Unlike the teacher model, due to the student model having a lesser end-to-end architecture and complexity, it used a lower learning rate to prevent inadequate learning within 30 epochs.

**Table 4:** Knowledge distillation hyper-parameters.

KD Hyper-parameter	Value
*τ*	2
*α*	0.3
Learning Rate	0.001

**Fig. 9:**
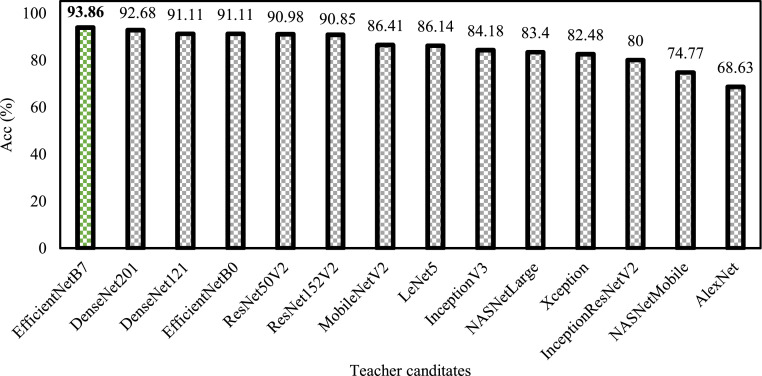
The overall performance of each teacher candidate trained on the mosquito dataset.

As mentioned, several teacher candidates underwent training to become the teacher model. Therefore, this method trained numerous SOTA models utilizing the previously introduced hyper-parameters with the prepared mosquito dataset. Fig. 9 reveals that the EfficientNetB7 became the predominant model that accomplished the automated taxonomy of the six mosquito species. Considering the number of recent and classical SOTA models involved, the EfficientNetB7, with an overall *Acc* of 93.86%, made it the ideal teacher model for the task.

Using standard metrics for measuring a DL model's performance, Table 5 presents the performance of the KD model that received the distilled knowledge from the EfficientNetB7 model during KD. Based on the calculated results, the model trained with this method performed best with a 100% *Acc* on the non-vector class while having the lowest performance of 99.61% on the *Aedes vexans, Anopheles sinensis, Culex tritaeniorhynchus* classes*.*

**Table 5:** Overall performance of the model trained using the proposed method.

Class	Validation Samples	*Acc*	*Pr*	*Rc*	*F1*
Non-Vector	120	100%	100%	100%	100%
*Aedes albopictus*	120	99.74%	100%	98.33%	99.16%
Aedes vexans	118	99.61%	97.52%	100%	98.74%
Anopheles sinensis	108	99.61%	100%	97.22%	98.59%
Culex pipiens	180	99.87%	100%	99.44%	99.72%
*Culex tritaeniorhynchus*	119	99.61%	97.54%	100%	98.76%
**Overall**	765	99.22%	99.24%	99.22%	99.22%

**Fig. 10:**
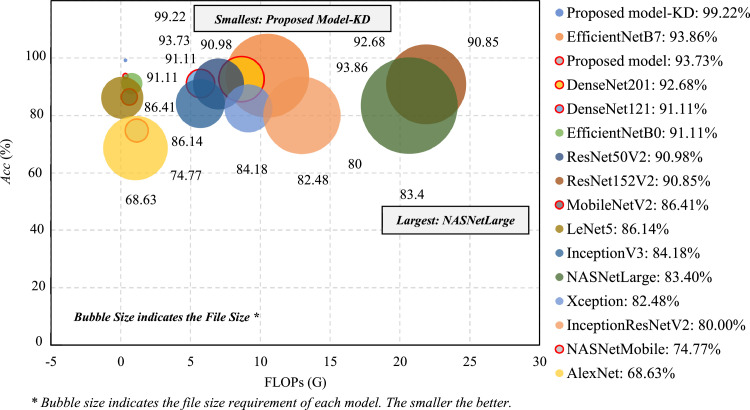
Comparison of various models’ performance to the cost ratio trained with the mosquito dataset.

After presenting this method's performance in automating mosquito species taxonomy, it is worth comparing it to a comprehensive list of SOTA models based on overall *Acc*, FLOPs, and disk size consumption [38]. As visualized in Fig. 10, the model trained with the proposed method achieved the highest overall *Acc* of 99.22%. Though it did not attain the lowest FLOPs, it still presents the best cost-to-performance efficiency based on its overall *Acc and* disk consumption of only 437 KB. Though LeNet5 had the lowest 0.11 GFLOPs, it only had an overall *Acc* of 86.14% and still consumes about 63 MB of disk space. On the other hand, the model produced from this method without KD also had a remarkable performance. The non-KD model's performance attained a 93.73% *Acc*, outperforming all the SOTA models except for the teacher model, EfficientNetB7, which had 93.86% *Acc*.

## Conclusion

Putting more awareness on the deployment and cost-efficiency of DL models can make them more adaptable and accessible even in the least fortunate areas. In this article, a proposed method of performing model compression on a SOTA model like EfficientNet highly reduced its overall cost. Though it had adverse effects, this method alleviated the lost feature generators by duplicating the compressed model and performing a layer-wise feature fusion. With the sense of potential overfitting from the shorter network and robust flowing features, this method also incorporated residual learning and self-normalization in the form of the MRSB activated by SeLU for added regularization. Based on the results, the model generated from this method trained with six mosquito species attained an overall performance of 99.22% *Acc*. In addition, it only consumes 437 KB of disk space and has a remarkable efficiency, as it only operates with 0.33 GFLOPs.

In conclusion, it shows that the proposed method has better potential to solve the difficulty in mosquito taxonomy better than most SOTA models that had FT and TL due to its less reliance on massive computing requirements. In addition, this article also exemplifies the proposed method's simplicity in yielding a lightweight and rich KD model. Future research can use and evaluate the method for other computer vision problems.

## Declaration of competing interests

The authors declare that they have no known competing financial interests or personal relationships that could have appeared to influence the work reported in this paper.

## Data Availability

The codes and dataset are included in the article.
